# Analysis of inorganic arsenic and methylarsenic in soil after derivatization by gas chromatography-mass spectrometry

**DOI:** 10.1371/journal.pone.0313924

**Published:** 2024-11-21

**Authors:** Wenzhi Zhao, Yuan Yang, Jintao Zhang, Tao Liu

**Affiliations:** 1 Center for Harbin Natural Resources Comprehensive Survey, China Geological Survey, Harbin, P. R. China; 2 Observation and Research Station of Earth Critical Zone in Black Soil, Ministry of Natural Resources, Harbin, P. R. China; University of Sindh, PAKISTAN

## Abstract

Gas chromatography-mass spectrometry (GC-MS) has been applied to the analysis of arsenic forms in water, plants, and other samples; however, it has not been used to determine the form of arsenic in soil due to the complex soil matrix. The purpose of this study was to develop an analytical method for the simultaneous determination of inorganic arsenic species (As (III) and As (V)) and monomethylarsonic acid (MMA) in soil using GC-MS. The arsenic compounds were subjected to derivatization with 2,3-dimercapto-1-propanol (BAL) and subsequently analyzed using GC-MS. The BAL volume, derivatization reaction time, and temperature were optimized using standard added soil extracts. A reaction with 150 μL of BAL at 40°C for 30 min was selected as the optimal condition for quantitative derivatization of both inorganic arsenic (iAs) and MMA. The calibration curves exhibited linearity within the range of 5–100 ng/mL for each arsenic species, with correlation coefficients exceeding 0.997. The limits of detection for each arsenic species were determined to be 0.24 ng/mL and 1.31 ng/mL, respectively. The accuracy of the method was verified by the recovery of reference samples. The recovery experiments for reference samples showed that the recovery rates for As (III), As (V), and MMA were 89.5–93.7%, 88.5–105.6%, and 90.2–95.8% respectively, with precision ranging from 4.6 to 6.5%, 2.3 to 3.8%, and 2.4 to 6.3%. These results indicate good accuracy and precision. The accuracy of this method is not significantly different from that of liquid chromatography-inductively coupled plasma mass spectrometry (p = 0.05). The optimized method was sensitive, convenient and reliable for the extraction and analysis of different arsenic species in soil samples.

## 1 Introduction

Arsenic is a toxic element that is widely distributed in the natural environment. Due to industrial development and the overuse of organic arsenic pesticides, soil pollution from arsenic in some agricultural products has gradually intensified. Arsenic is easily absorbed by plants and accumulates in their bodies, posing a potential threat to human health [1,2]. Arsenic pollution has become a global environmental issue. Arsenic exists in various forms in the environment, with the compound form mainly divided into two categories: inorganic arsenic and organic arsenic. Inorganic arsenic includes arsenite (As (Ⅲ)) and arsenate (As (Ⅴ)), while organic arsenic mainly consists of monomethylarsenate (MMA), dimethylarsenate (DMA), arsenic betaine, arsenic choline, and arsenic sugar [3]. The toxicity of different forms of arsenic varies greatly, with inorganic arsenic typically being over 100 times more toxic than organic arsenic. The International Agency for Research on Cancer has confirmed that inorganic arsenic and its compounds are classified as Group 1 carcinogens. The form of arsenic determines the mobility, migration, and transformation in soil, as well as the degree of absorption and utilization by organisms [4–6]. The study of total arsenic content alone cannot accurately and comprehensively evaluate the extent of arsenic pollution in the environment, making it particularly important to analyze the form of arsenic in soil [7,8].

The main methods currently used for analyzing arsenic forms are liquid chromatography-atomic fluorescence spectrometry (LC-AFS) [9–14] and liquid chromatography-inductively coupled plasma mass spectrometry (LC-ICP-MS) [15–21]. LC-AFS technology is the most commonly used method for arsenic form analysis, which has a low testing cost but high requirements for the mobile phase. Additionally, it exhibits poor long-term stability of the instrument and low sensitivity, making it unsuitable for analyzing samples with low content [22,23]. The LC-ICP-MS technique has a low detection limit, high sensitivity, and a wide linear range of analysis. However, it is complicated to operate, expensive to use, and has high testing costs [24,25]. Additionally, due to the lack of reference materials, it is impossible to detect and characterize unknown forms of arsenic.

Gas chromatography-mass spectrometry (GC-MS) combines the advantages of high efficiency separation of GC and low detection limit and wide dynamic linear range of MS. It has a high analytical speed and sensitivity in determining thermally stable and volatile samples [26]. Therefore, different forms of arsenic can be converted into weakly polar, highly stable, and volatile arsenic derivatives through derivatization methods. These arsenic derivatives can be analyzed by GC-MS to achieve good chromatographic separation and high detection sensitivity, meeting the requirements for morphological detection of arsenic. Previously, GC-MS has been employed for arsenic speciation through derivatization with various thiols such as thioglycol methylate (TGM), thioglycol ethylate (TGE), and BAL, which are widely recognized reagents for the analysis of arsenic compounds [27]. The reaction of inorganic arsenic compounds is not selective, which means that both As (III) and As (V) react with the derivatizing agent to form the same derivative [28]. In other words, this technology can only measure the total content of iAs. From the legislative and soil safety perspectives, complete speciation of arsenic species is deemed unnecessary. Even distinguishing between the two inorganic arsenic species, As (III) and As (V), holds little significance [29]. Currently, this technology is primarily applied to the analysis of water [28], plants [25,29,30] and other samples [27,31]. One study has also reported the use of GC for determining the total arsenic content in soil [32]; however, GC-MS has not been employed to determine the form of arsenic in soil samples. Therefore, there is an urgent need for fast, sensitive, selective, and cost-effective methodologies to analyze arsenic species in soil.

The objective of this study was to establish a GC-MS method for simultaneous determination of iAs and MMA in soil samples. In order to achieve the required sensitivity, we systematically screened the most efficient derivatives and optimized the derivatization conditions. The accuracy of the method was verified using reference soil samples, while actual soil samples were used to confirm its feasibility.

## 2 Methodology

### 2.1 Instrument

The arsenic analytes were qualitatively and quantitatively analyzed using an GC-MS (QP2020, Shimadzu, Tokyo, Japan). The Avanti J-E centrifuge (Beckman Coulter, Germany) and M5800-J ultrasonic cleaner (Bransonic, USA) were employed for centrifugation and ultrasound-assisted extraction, respectively. The operating parameters of GC-MS were shown in **Table 1**.

**Table 1:** Operating parameters of GC-MS.

Item	Parameter
chromatographic column	Rts-5SiMS (30 m×0.25 mm×0. 25 μm)
oven temperature	60°C_20°C/min_220°C (1 min)_5°C/min_280°C (4 min)
the temperatures of the injection port	250°C
injection volume	1.0 μL
Injection Mode	Splitless (1 min)
carrier gas/flow rate	Helium/1.0 mL/min
electron energy	70 eV
ion source	220°C
scan mode	m/z 100–300
ion monitoring (SIM)	iAs-BAL (m/z 212, 165, 107); MMA-BAL (m/z 212, 197, 107)

### 2.2 Reagents, solutions and samples

Stock solutions were prepared from standard substances sodium arsenite, sodium arsenate, monomethylarsonic acid (MMA) and dimethylarsinic acid (DMA) (Sinopharm, Beijing, China). All aqueous standards (1 mg L^-1^) were made by using ultrapure water and stored at 4°C for not more than two weeks. As internal standard (IS) hexachlorobenzene (Sinopharm, Beijing, China) dissolved in cyclohexane was applied.

The derivatization reagents for As (III), As (V), MMA and DMA were BAL, TGM, TGE, 1,3-propanedithiol (PDT) and 1,5-pentanedithiol (PeDT) (Sinopharm, Beijing, China). Hydrochloric acid (HCl), tin (II) chloride dihydrate (SnCl_2_.2H_2_O), potassium iodide (KI), dichloromethane (DCM) were purchased from Sinopharm Chemical Reagent Co. Ltd, China. SnCl_2_ solution was prepared by dissolving SnCl_2_.2H_2_O in 50 mg/ml HCl. KI and BAL were dissolved in water to prepare 20% (w/w) KI solution and 0.2% (v/v) BAL solution. The solutions were all prepared using high purity water (18.2 MΩ cm resistivity) obtained from the GN-RO-500 Total Water System (Shuangfeng, Beijing, China).

Validation experiments were carried out using the following reference soil samples: GSS31, GSS32, GSS33, GSS34, (soil, IGGE, China). The selection of these samples was based on the required concentration interval and the quality of available data for each reference material. Center for Harbin Natural Resources Comprehensive Survey has permitted the work and provided relevant analytical instruments, reagents, and soil samples.

### 2.3 Sample extraction

The soil sample weighing 0.20 g was accurately placed into a 50 mL polypropylene tube. Then, 25 mL of a mixed solution containing phosphoric acid and ascorbic acid with concentrations of 1.0 mol/L and 0.1 mol/L respectively was added to the tube. The mixture was heated in a water bath at 100°C for 3 h. After cooling, the supernatant was centrifuged at a speed of 5000 r/min for 20 min and filtered through an organic filter membrane with a pore size of 0.45 μm.

### 2.4 Derivatization

As previously reference described [33], 1 mL of sample extract or standard solution was transferred into a 15 mL polypropylene tube. The tube was subjected to vortex-mixing for 10 s after the addition of SnCl_2_ (0.4 mL) and KI (0.2 mL) solutions, followed by a 30 min incubation at room temperature.

For the derivatization of BAL [29], 150 μL of BAL solution was added to the tube and placed in a water bath at 40°C for 30 min. When performing the extraction of arsenic derivatives, a mixture consisting of 1 mL of dichloromethane and 10 μL of a hexachlorobenzene solution (1 mg/mL) was introduced into the designated tube. After being agitated for a duration of 2 min, the organic phase of the reaction mixture was subjected to direct analysis using GC-MS.

For the derivatization of TGM and TGE [31], the pH was adjusted to 2 by the addition of 0.28M HNO_3_, followed by the subsequent addition of 25 μL of either TGM or TGE. The mixture was gently agitated for a minimum of 2 min prior to the addition of 1 mL of cyclohexane and 10 μL of hexachlorobenzene (dissolved in cyclohexane at a concentration of 1 μg/mL). After vortexing for an additional 2 min, the organic phase of the reaction mixture was subjected to direct analysis using GC-MS.

For the derivatization of PDT and PeDT [31], the mixture was heated to 70°C after the addition of 500 μL of 5 M HCl, followed by the addition of either PDT or PeDT (2 μL). The reaction proceeded for 5 min before being cooled to room temperature. Subsequently, 1 mL of toluene and 10 μL of hexachlorobenzene dissolved in cyclohexane (at a concentration of 1 μg/mL) were sequentially added. After vortexing for an additional 2 min, the organic phase of the reaction mixture was subjected to direct analysis using GC-MS.

### 2.5 Statistical analysis

The statistical significance of the iAs and MMA contents was assessed using Analysis of Variance (ANOVA) and Duncan’s multiple-range test at a significance level of P = 0.05, employing SPSS 18.0 software (IBM Inc., USA).

## 3 Results and discussion

### 3.1 Comparison of the derivatization reagents

**Fig 1:**
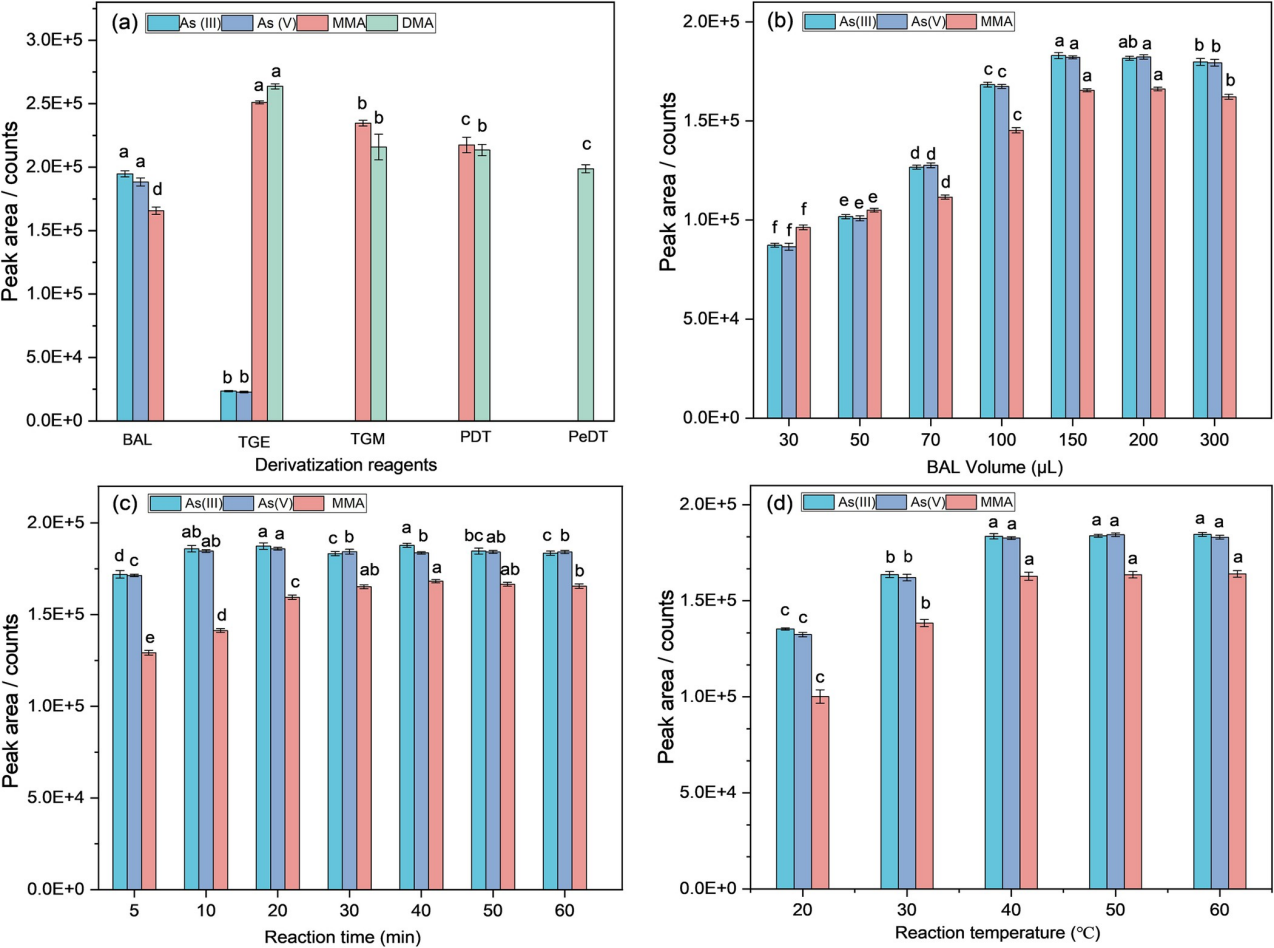
Optimization of reaction conditions (a: Derivatization reagents, BAL volume 150 μL, reaction time 30 min, reaction temperature 40°C; b: BAL volume, reaction time 30 min, reaction temperature 40°C; c: Reaction time, BAL volume 150 μL, reaction temperature 40°C; d: Reaction temperature, BAL volume 150 μL, reaction time 30 min; The error bars represent the standard deviation of the recovery rate for each element (n = 7). Statistical analysis was carried out by ANOVA and Duncan’s multiple range test. The different letter in each bar graph denotes that means are significant different at ɑ = 0.05).

For the choice of an optimal derivatizing reagent, we examined the use of BAL, TGM, TGE, PDT and PeDT. The standard soil extract was prepared in a 0.28 M HNO3 solution, containing 1 μg/mL of each As (III), As (V), MMA, and DMA, respectively. As depicted in **Fig 1A**, the derivative reaction of PeDT exclusively yielded the signal of MMA-PeDT. The detection and identification of both organometallic derivatives were achieved through mass spectrometry in the TGM and PDT reactions. For the derivatization of TGE, all arsenic analytes were present in the chromatogram. Compared to other derivatization reagents, MMA-TGE and DMA-TGE exhibited the highest signal intensity. However, the sensitivity of iAs derivatives was significantly low, with iAs only detectable within a concentration range of μg/mL in the chromatogram. This observation aligns with the research findings reported by Richter et al [31]. It is plausible that this limited sensitivity could be attributed to either incomplete reaction between iAs and TGE or its potential decomposition subsequent to injection into the GC system. Compared to these derivatization reagents, the derivatization reaction of BAL has the best effect on the derivatization and chromatographic separation of iAs and MMA, although no chromatographic signal of DMA-BAL was observed. Therefore, we have chosen BAL as the derivatizing reagent.

### 3.2 Effect of the BAL volume

The reaction between arsenic analytes and BAL, as well as the exposure of the derivatizing agent to air, leads to the formation of BAL-dimers. This compound, along with an excess amount of BAL, may interfere with the capillary GC column or affect the GC-MS system [31]. To mitigate these adverse effects, it is recommended to use minimal amounts of BAL for derivatization.

To determine the optimal quantity of BAL solution for iAs and MMA derivations in soil samples, varying volumes ranging from 30 to 300 μL of BAL solution were introduced into soil extract containing 1 μg/mL each of As (III), As (V), and MMA. The standard soil extract was then conducted at the selected reaction temperature of 40°C and for a duration of 30 min. As depicted in **Fig 1B**, no significant disparity was observed in the response of As (III) and As (V) to BAL at any volume (P > 0.05), indicating complete conversion of As (V) into As (III) during the reaction with SnCl_2_ and KI. Upon reaching a BAL volume of 150 μL, iAs-BAL and MMA-BAL attained their optimal peaks.

### 3.3 Effect of reaction time and temperature

The reaction temperature and duration were identified as crucial factors influencing the derivatization reaction of both iAs and MMA [29]. In order to shorten time of the sample preparation, the extraction time was studied in the range of 5~60 min. The results showed that there was no significant difference in the derivatization peak of iAs-BAL after 10 min, while the peak of MMA-BAL showed no significant difference after 30 min (P > 0.05) (**Fig 1C**).

When the reaction time was constant, the derivative peak area of iAs and MMA increased with increasing temperature, especially for MMA. A significant increase in the derivative peak area of MMA-BAL was observed. The peaks of iAs-BAL and MMA-BAL tend to stabilize when the temperature reached 40°C (**Fig 1D**). Compared to iAs, the reaction of MMA and BAL was more dependent on temperature and time. Therefore, the reaction condition (40°C and 30 min) was selected as the optimal derivatization for quantitatively deriving both iAs and MMA.

### 3.4 Analytical validation characteristics

**Fig 2:**
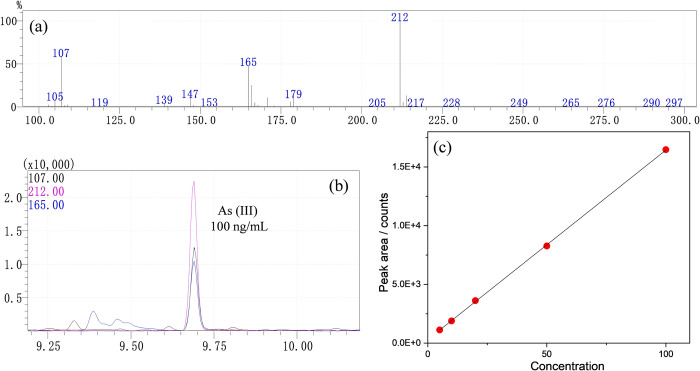
Representative Mass spectrum (a) and gas chromatograms (b) for standard As (III) solutions (100 ng/mL). MS calibration models of As (III) (c) in a concentration range of 5–100 ng/mL.

The mass spectrometric characteristic ions, calibration curves, correlation coefficients, concentration ranges, LODs, and LOQs of iAs-BAL and MMA-BAL were presented in **Table 2** and **Fig 2**. The analysis was conducted using five concentration levels containing As (III), As (V), and MMA. Within the concentration range of 5–100 ng/mL, a strong linear relationship was observed among the three analytes, with a correlation coefficient exceeding 0.9973.

**Table 2:** Analytical processes characteristics of iAs and MMA analysis using GC-MS method.

Analytes	Molecular fragment ions (m/z)	Calibration curve	R^2^	Test range (ng/mL)	LOD (ng/mL)	LOQ (ng/mL)
As (III)	212 (C_3_H_5_0_2_S_2_As), 165 (C_2_H_2_S_2_As^+^), 107 (AsS^+^)	y = 162.10x+252.54	0.9998	5–100	0.24	0.8
As (V)	212 (C_3_H_5_0_2_S_2_As), 165 (C_2_H_2_S_2_As^+^), 107 (AsS^+^)	y = 116.17x-28.83	0.9992	5–100	0.24	0.8
MMA	212 (C_4_H_9_0S_2_As^+^), 197 (C_3_H_6_0S_2_As^+^), 107 (AsS^+^)	y = 110.81x-171.08	0.9973	5–100	1.31	4.4

The LOD and LOQ are defined as the amounts injected that give signals equivalent to 3 and 10 times the baseline noise, respectively [29]. The LODs of As (III), As (V) and MMA were 0.24, 0.24, and 1.31 ng/mL, while the LOQs were 0.8, 0.8, and 4.4 ng/mL. Additionally, the reliability of the analysis procedure was determined through recovery experiments. Specifically, 5 μg/g of As (III), As (V) and MMA were added to reference soil samples GSS31-GSS34 and subsequently analyzed. The average measured value (n = 7), recoveries, and relative standard deviation (%RSD) (n = 7) were determined for each reference soil sample and presented in **Table 3**. The recoveries of As (III), As (V), and MMA were found to be within the range of 89.5–93.7%, 88.5–105.6%, and 90.2–95.8% respectively, indicating excellent accuracy in the analytical measurements conducted. Additionally, the RSD for these analytes ranged from 4.6 to 6.5%, 2.3 to 3.8%, and 2.4 to 6.3% respectively, demonstrating a high level of precision.

**Table 3:** Determination of arsenic analytes in reference soil samples using the proposed methods.

Sample	Elements	Background values (mg/kg)	Spiked level (mg/kg)	Measured (mg/kg)	Recovery (%)	RSD (%)
GSS 31	As (III)	0.71±0.05	5.00	5.11±0.37	89.5	4.6
	As (V)	4.67±0.38	5.00	10.21±0.93	105.6	3.5
	MMA	—	5.00	4.57±0.41	91.4	6.3
GSS 32	As (III)	2.81±0.35	5.00	7.07±1.1	90.5	6.5
	As (V)	6.58±0.43	5.00	11.03±0.88	95.3	3.8
	MMA	—	5.00	4.51±0.39	90.2	3.1
GSS 33	As (III)	3.42±0.28	5.00	7.73±0.67	91.8	5.5
	As (V)	6.61±0.54	5.00	11.14±0.91	96.0	2.3
	MMA	—	5.00	4.79±0.42	95.8	2.4
GSS 34	As (III)	0.25±0.02	5.00	4.92±0.37	93.7	5.2
	As (V)	9.98±0.77	5.00	13.25±0.93	88.5	2.3
	MMA	—	5.00	4.64±0.28	92.8	4.7

Values are for mean±standard deviation of seven replicate measurements (n = 7).

**Fig 3:**
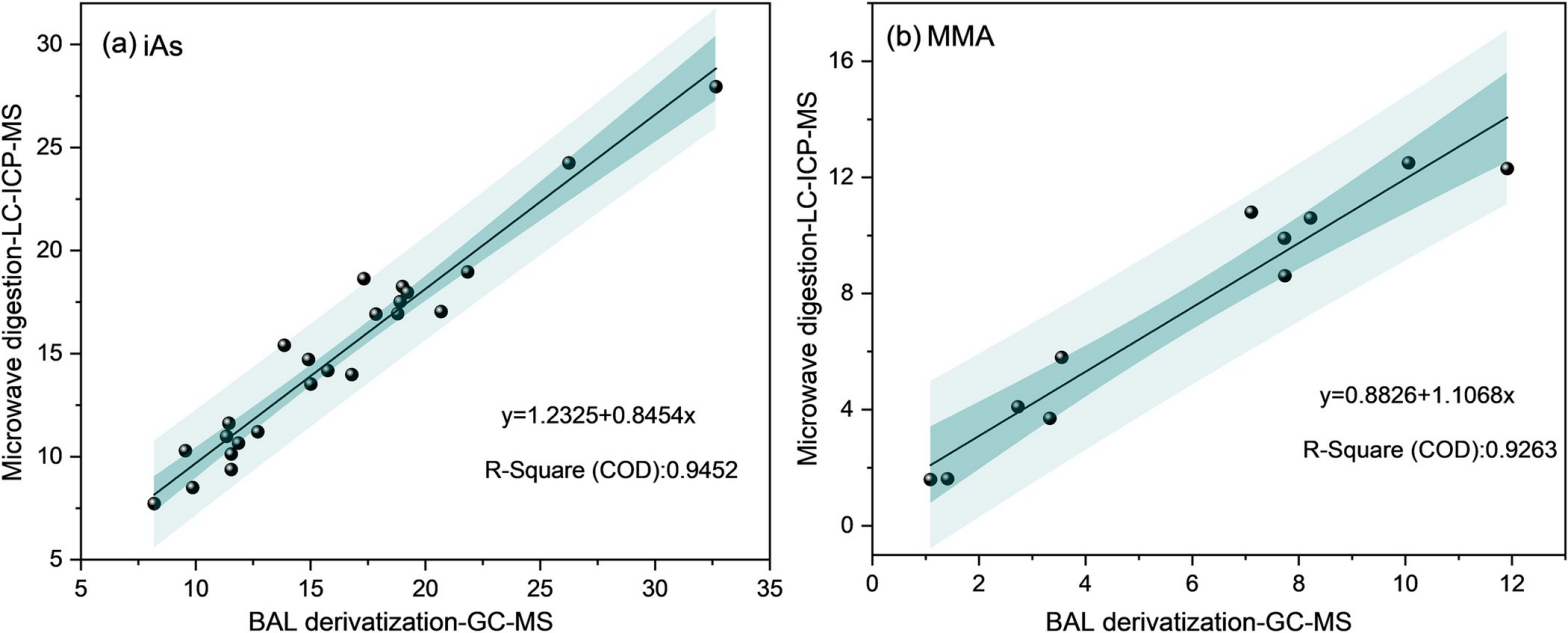
Correlation between measured values obtained from the developed and reference methods.

The proposed method was also employed to determine the iAs content in a total of 24 actual soil samples, including 8 haplic phaeozem samples, 8 brown soil samples, and 8 chernozem samples. Furthermore, this method was applied to analyze the concentration of MMA in a set of 11 sludge specimens. The determination of arsenic forms by liquid chromatography-inductively coupled plasma mass spectrometer (LC-ICP-MS) is widely recognized as a robust analytical technique [33], thus making it the designated reference method for this study. The proposed method shows strong agreement with the reference method, supported by high correlation coefficients (R^2^) of 0.9452 and 0.9263 for iAs and MMA determination, respectively, as depicted in Fig 3. Thus, this study effectively validates the utilization of GC-MS analysis as an efficient approach to determine various forms of arsenic in real soil samples.

## 4 Conclusions

The present study employed GC-MS to establish a robust method for the simultaneous determination of iAs and MMA in soil samples. Various derivatization methods were assessed for their efficacy in analyzing different arsenic species, ultimately identifying BAL as the most effective derivatization reagent. The derivatization process has been optimized, resulting in a good linear relationship over a wide concentration range, low LODs and LOQs, good accuracy and high precision. The developed method for determining arsenic analytes in various soil samples was successfully validated through method verification. A comparative analysis between the proposed method and the LC-ICPMS method revealed no significant difference in accuracy, thereby confirming the reliability of this approach. The proposed method demonstrated high sensitivity, convenience and reliability for the extraction and analysis of diverse arsenic species in soil samples.

Although GC-MS technology has been widely used in the analysis of arsenic species, several problems have limited the development of this method. (1) Inability to distinguish between As(III) and As(V); (2) Although there are various types of derivatives, each derivative possesses inherent limitations; for instance, determining DMA can be challenging in the case of BAL; (3) The soil composition is complex, and there is a lack of reference soils for testing analytes. The absence of reference soil makes it difficult to identify the chemical structure of the unknown compound. The aforementioned issues can serve as the guiding direction for future research and development.

## Supporting information

S1 Fig Derived equations of different arsenic species and BAL. (TIF)

S2 Fig The main cleavage pathway of iAs-BAL derivatives. (TIF)

S3 Fig Representative Mass spectrum (a) and gas chromatograms (b) for standard As (V) solutions. MS calibration models of As (V) (c) in a concentration range of 5–100 ng/mL.
(TIF)

S4 Fig Representative Mass spectrum (a) and gas chromatograms (b) for standard MMA solutions. MS calibration models of MMA (c) in a concentration range of 5–100 ng/mL.
(TIF)

S1 Table Determination of iAs in actual samples by LC-ICP-MS and GC-MS. (DOC)

S2 Table Determination of MMA in actual samples by LC-ICP-MS and GC-MS. (DOC)

## Abbreviations

iAs: inorganic arsenic
MMA: monomethylarsonic acid
DMA: dimethylarsinic acid
BAL: 2,3-dimercapto-1-propanol
TGM: thioglycol methylate
TGE: thioglycol ethylate
PDT: 1,3-propanedithiol
PeDT: 1,5-pentanedithiol
GC-MS: gas chromatography-mass spectrometry
LC-ICP-MS: liquid chromatography-inductively coupled plasma mass spectrometry
