# Supplementary figures and images for: Analysis of inorganic arsenic and methylarsenic in soil after derivatization by gas chromatography-mass spectrometry
Source: PLoS One. 2024 Nov 21;19(11):e0313924. doi: 10.1371/journal.pone.0313924 (PMC11581245; doi:10.1371/journal.pone.0313924)

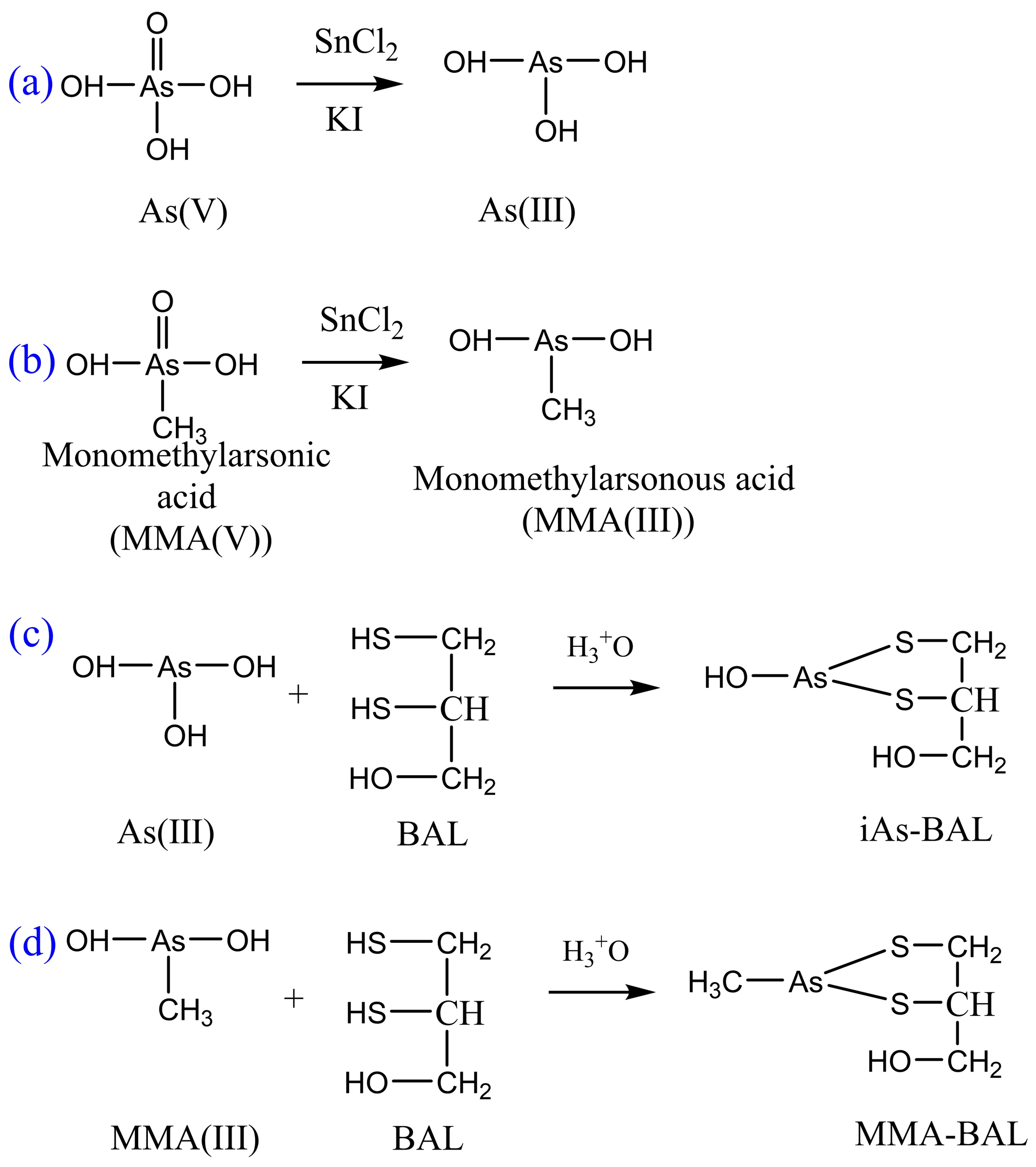

Supplement: S1 Fig — (TIF) [file pone.0313924.s001.tif]

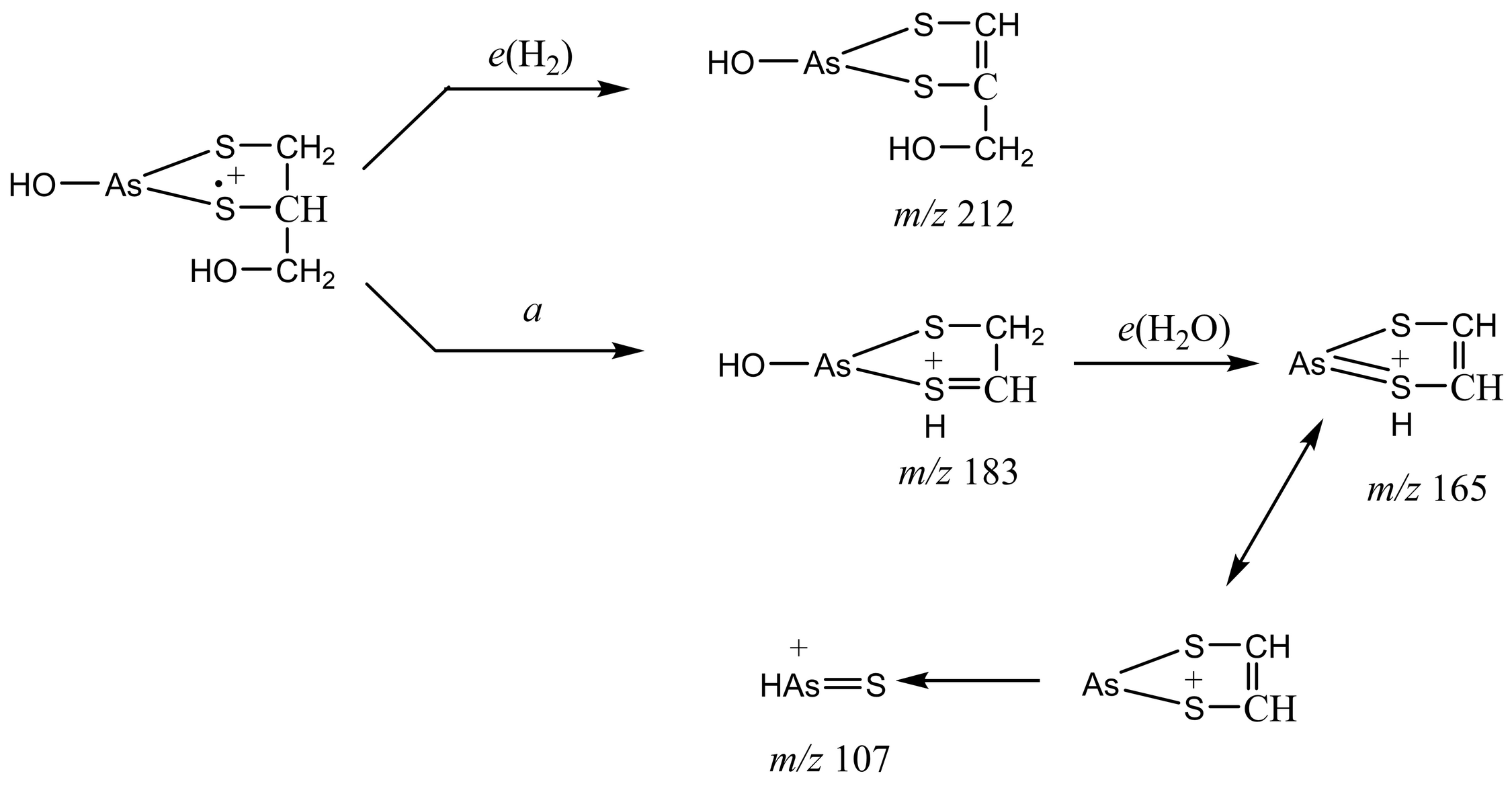

Supplement: S2 Fig — (TIF) [file pone.0313924.s002.tif]

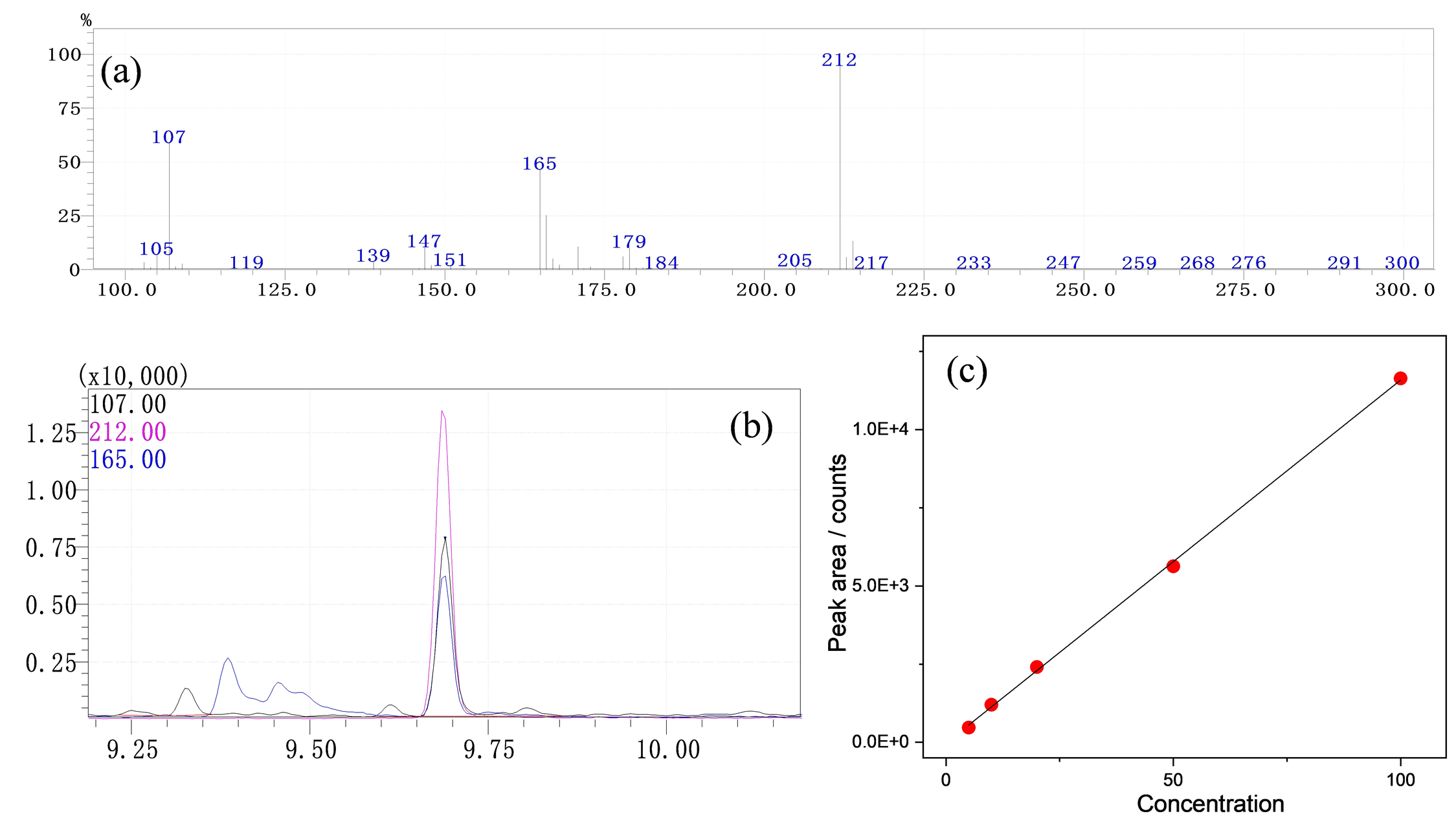

Supplement: S3 Fig — Representative Mass spectrum (a) and gas chromatograms (b) for standard As (V) solutions. MS calibration models of As (V) (c) in a concentration range of 5–100 ng/mL. (TIF) [file pone.0313924.s003.tif]

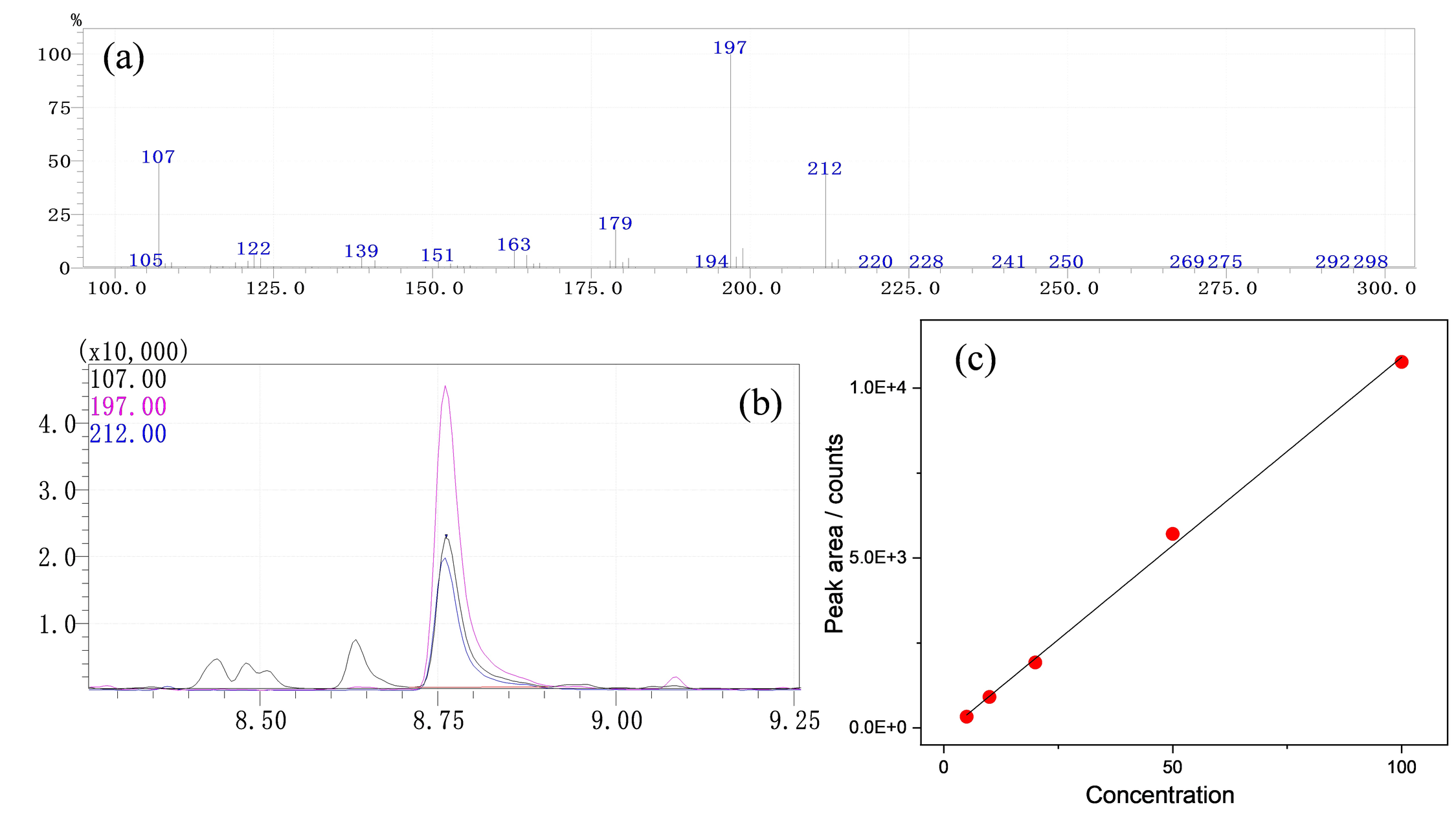

Supplement: S4 Fig — Representative Mass spectrum (a) and gas chromatograms (b) for standard MMA solutions. MS calibration models of MMA (c) in a concentration range of 5–100 ng/mL. (TIF) [file pone.0313924.s004.tif]
