# Supplementary material for: Analysis of inorganic arsenic and methylarsenic in soil after derivatization by gas chromatography-mass spectrometry
Source: PLoS One. 2024 Nov 21;19(11):e0313924. doi: 10.1371/journal.pone.0313924 (PMC11581245; doi:10.1371/journal.pone.0313924)
Supplement: S1 Table — (DOC) [file pone.0313924.s005.doc]

**Table1s**. Determination of iAs in actual samples by LC-ICP-MS and GC-MS

| Sample | Microwave digestion-LC-ICP-MS  /(mg kg^−1^) | BAL derivatization-GC-MS  /(mg kg^−1^) |
| --- | --- | --- |
|
| S1 (haplic phaeozem) | 19.01±1.26 | 18.25±1.35 |
| S2 (haplic phaeozem) | 20.69±1.33 | 17.03±1.42 |
| S3 (haplic phaeozem) | 12.71±0.87 | 11.21±0.90 |
| S4 (haplic phaeozem) | 15.02±0.96 | 13.52±1.05 |
| S5 (haplic phaeozem) | 13.86±0.91 | 15.40±1.33 |
| S6 (haplic phaeozem) | 18.80±1.22 | 16.94±1.27 |
| S7 (haplic phaeozem) | 21.84±1.37 | 18.96±1.55 |
| S8 (haplic phaeozem) | 32.66±4.31 | 27.95±2.11 |
| S9 (brown soil) | 11.45±0.97 | 11.62±1.14 |
| S10 (brown soil) | 9.87±1.05 | 8.50±0.91 |
| S11 (brown soil) | 8.19±0.95 | 7.72±0.96 |
| S12 (brown soil) | 11.34±0.96 | 10.98±1.05 |
| S13 (brown soil) | 14.91±1.14 | 14.72±1.11 |
| S14 (brown soil) | 9.56±1.12 | 10.29±0.99 |
| S15 (brown soil) | 19.22±1.35 | 17.97±1.42 |
| S16 (brown soil) | 17.33±1.32 | 18.63±1.37 |
| S17 (chernozem) | 11.55±0.93 | 10.12±0.98 |
| S18 (chernozem) | 18.90±1.33 | 17.52±1.51 |
| S19 (chernozem) | 26.25±2.58 | 24.25±2.05 |
| S20(chernozem) | 16.80±1.42 | 13.98±1.46 |
| S21 (chernozem) | 15.75±1.18 | 14.18±1.29 |
| S22 (chernozem) | 11.55±0.95 | 9.37±1.07 |
| S23 (chernozem) | 17.85±1.23 | 16.91±1.28 |
| S24 (chernozem) | 11.87±1.08 | 10.66±0.97 |

Values are for mean±standard deviation of seven replicate measurements (n=7)
