# Supplementary material for: Analysis of inorganic arsenic and methylarsenic in soil after derivatization by gas chromatography-mass spectrometry
Source: PLoS One. 2024 Nov 21;19(11):e0313924. doi: 10.1371/journal.pone.0313924 (PMC11581245; doi:10.1371/journal.pone.0313924)
Supplement: S2 Table — (DOC) [file pone.0313924.s006.doc]

**Table2s**. Determination of MMA in actual samples by LC-ICP-MS and GC-MS

| Sample | Microwave digestion-LC-ICP-MS  /(mg kg^−1^) | BAL derivatization-GC-MS  /(mg kg^−1^) |
| --- | --- | --- |
|
| S1 (sludge) | 10.06±1.11 | 12.5±1.23 |
| S2 (sludge) | 7.11±0.95 | 10.8±1.05 |
| S3 (sludge) | 7.73±1.02 | 9.9±0.95 |
| S4 (sludge) | 2.73±0.17 | 4.1±0.38 |
| S5 (sludge) | 3.55±0.38 | 5.8±0.61 |
| S6 (sludge) | 11.91±1.03 | 12.3±1.12 |
| S7 (sludge) | 8.22±0.81 | 10.6±1.18 |
| S8 (sludge) | 3.33±0.45 | 3.7±0.541 |
| S9 (sludge) | 1.09±0.09 | 1.59±0.23 |
| S10 (sludge) | 1.41±0.19 | 1.62±0.21 |
| S11 (sludge) | 7.74±0.87 | 8.61±0.94 |

Values are for mean±standard deviation of seven replicate measurements (n=7)
